# Respiratory *Aspergillus* Colonization Was Associated With Relapse of Acute Exacerbation in Patients With Chronic Obstructive Pulmonary Disease: Analysis of Data From A Retrospective Cohort Study

**DOI:** 10.3389/fmed.2021.640289

**Published:** 2021-05-04

**Authors:** Yi-xing Wu, Yi-hui Zuo, Qi-jian Cheng, Yi Huang, Zhi-yao Bao, Xiao-yan Jin, Xi-wen Gao, Chun-lin Tu, Wei-ping Hu, Jing-qing Hang, Wei-qin Wang, Feng-ying Zhang, Jing Zhang

**Affiliations:** ^1^Department of Pulmonary and Critical Care Medicine, Zhongshan Hospital, Fudan University, Shanghai, China; ^2^Department of Respiratory Medicine, Ruijin North Hospital, School of Medicine, Shanghai Jiao Tong University, Shanghai, China; ^3^Department of Pulmonary and Critical Care Medicine, Changhai Hospital of Shanghai, Second Military Medical University, Shanghai, China; ^4^Department of Respiratory Medicine, Ruijin Hospital, School of Medicine, Shanghai Jiao Tong University, Shanghai, China; ^5^Department of Respiratory Medicine, Tongren Hospital, School of Medicine, Shanghai Jiao Tong University, Shanghai, China; ^6^Department of Respiratory Medicine, Central Hospital of Minhang District, Shanghai, Fudan University, Shanghai, China; ^7^Department of Respiratory Medicine, Central Hospital of Jiading District, Shanghai, Fudan University, Shanghai, China; ^8^Department of Respiratory Medicine, Shanghai Putuo District People's Hospital, Shanghai, China

**Keywords:** chronic obstructive pulmonary disease, acute exacerbation, *Aspergillus*, prognosis, recurrence of AECOPD

## Abstract

**Background:** Patients with chronic obstructive pulmonary disease (COPD) are more susceptible to *Aspergillus* colonization or infection. Several studies have demonstrated that invasive pulmonary Aspergillosis (IPA) and *Aspergillus* hypersensitivity (AH) have a detrimental effect on COPD. However, it remains to be clarified whether *Aspergillus* colonization is associated with acute exacerbation of COPD (AECOPD). This study aimed to explore the impact of *Aspergillus* colonization in the lower respiratory tract on AECOPD.

**Method:** Patients with *Aspergillus* colonization were identified from a retrospective cohort of hospitalized AECOPD from 2011 to 2016 in eight centers in Shanghai, China. The demographic information, conditions of the stable stage, clinical characteristics during hospitalization, and 1-year follow-up information after discharge were collected and compared to participants without fungi colonization.

**Result:** Twenty-six hospitalized AECOPD patients with *Aspergillus* colonization and 72 controls were included in the final analysis after excluding patients with other fungi isolation and matching. The rates of recurrence of acute exacerbation within 90 days and 180 days after discharge in the patients with *Aspergillus* colonization were both significantly higher than that in the fungi negative patients (90 days: 19.2 vs. 4.2%, *p* = 0.029; 180 days: 23.1 vs. 4.2%, *p* = 0.010), and the all-cause mortality within 1 year was also higher (11.5 vs. 0.0%, *p* = 0.017). Multivariate logistic regression analysis showed that *Aspergillus* colonization was an independent risk factor for the recurrence of acute exacerbation within 90 days and 180 days (90 days: OR = 8.661, 95% CI: 1.496-50.159, *p* = 0.016; 180 days: OR =10.723, 95% CI: 1.936-59.394, *p* = 0.007).

**Conclusion:** *Aspergillus* colonization may predict poor prognosis of AECOPD while leading to an increased risk of recurrent AECOPD in a short period.

## Introduction

Chronic obstructive pulmonary disease (COPD) is a common chronic respiratory disease characterized by progressive and irreversible airflow limitation. Due to the aging population, the expanding epidemic of cigarette smoking, and other reasons, COPD's prevalence and mortality are among the highest worldwide while causing significant economic and social burden (1). Acute exacerbation of chronic obstructive pulmonary disease (AECOPD) refers to the deterioration of respiratory symptoms in a short period and requiring additional treatment. Exacerbation is the primary factor of poor outcomes of this disease, including loss of working capacity, a decline in lung function, hospitalization, and sometimes even death (2–4). AECOPD is mainly induced by pathogenic microorganisms and environmental factors. Bacteria and viruses are considered as the usual triggers for acute exacerbations rather than fungi.

*Aspergillus* is an airborne fungal pathogen that spreads all over the world. The conidia can reach the pulmonary parenchyma through the airway as its diameter is only 2-3 μm. Meanwhile, chronic airway inflammation in COPD results in damage to the epithelium of the lower respiratory tract (LRT) and further weakens the defense of the airways. As a result, COPD patients are susceptible to *Aspergillus* colonization or infection (5). *Aspergillus* usually colonize the airways without generating infection. It is reported that the prevalence of *Aspergillus* colonization in COPD patients is from 8.33 (6) to 16.6% (7). According to the host immune response, *Aspergillus* can cause invasive pulmonary aspergillosis (IPA), allergic bronchopulmonary aspergillosis (ABPA), or other clinical syndromes (8). Growing evidence support that COPD is the risk factor of IPA, which carries an abysmal prognosis (5, 9). A study between COPD patients and healthy volunteers demonstrated that COPD might predispose *Aspergillus* hypersensitivity (AH) and ABPA (10). It has been reported that allergy and AH are associated with severe symptoms and reduction of lung function in COPD patients (11, 12).

It is necessary to find out the significance of *Aspergillus* colonization in COPD patients for clinical decision making. But the effect of *Aspergillus* colonization in COPD remains to be studied and clarified, especially on the prognosis. Therefore, we carried out this study to investigate the impact of LRT *Aspergillus* colonization on the prognosis of patients hospitalized for AECOPD, which might help with the management strategies.

## Patients and Methods

### Study Design

This multicenter retrospective cohort study was initiated by the Department of Pulmonary and Critical Care Medicine, Zhongshan Hospital, Fudan University in Shanghai, China. We collected patients hospitalized with AECOPD from January 2011 to December 2016 in eight hospitals in Shanghai. From the cohort, those with LRT *Aspergillus* colonization were identified (*Aspergillus* positive group) and those without evidence of *Aspergillus* colonization were classified as controls (*Aspergillus* negative group). The LRT *Aspergillus* colonization was defined as culture positive for *Aspergillus* from samples including sputum, endotracheal suction, bronchoscopic flushing, and/or lavage fluid (BALF), and had no clinical presentation of fungal infection during hospitalization. Propensity score matching was applied if there was a significant difference in baseline characteristics between the two groups. The ethics committee of Zhongshan Hospital of Fudan University approved this study (Approval notice number: B2015-119R).

### Culture of *Aspergillus*

The microbiologic findings of LRT samples were carried out in the following way. After obtaining the sample from the patient, it was quickly sent to the microbiology laboratory at room temperature, and processed on the same day. About 10 μl samples were pipetted directly from the submitted specimen and inoculated onto sabouraud dextrose agar (SDA) containing chloramphenicol and incubated at 28°C~35°C for 7 days. The eight units that performed the *Aspergillus* culture were all under the quality control of Shanghai Center For Clinical Laboratory, and the examiners had all received professional training.

### Inclusion Criteria

Those with all the following criteria were enrolled: (1) Age over 18 years old. (2) The hospitalized patient met the diagnostic criteria for COPD and acute exacerbations in the 2017 GOLD report. (3) The patients' LRT samples had been detected for *Aspergillus*.

### Exclusion Criteria

Those who met any of the following items were excluded: (1) Chest CT findings suggested chronic pulmonary aspergillosis (CPA), IPA or ABPA. (2) A positive result of serum or BALF galactomannan determination. (3) Treatment of anti-fungal therapy. (4) A positive culture of other fungi except for *Aspergillus* from LRT specimens. (5) Sputum smears showed spores indicating *Candida*. (6) Patients with mental diseases that made themselves unable to cooperate in data collection. (7) Combined with other underlying lung diseases, such as lung cancer, bronchiectasis, interstitial pulmonary diseases, lung abscess, and tuberculosis. (8) Patients with immunocompromised diseases.

### Data Collection

The patients' clinical data who met the inclusion criteria through their hospitals' electronic and paper medical records were collected and put into the Epidata system (The EpiData Association, Odense, Denmark). An independent investigator would check the collected data. If inconsistent data were found, the examiner would correct it after checking the original case history.

The information to be collected was as follows: basic patient characteristics (sex, age, smoking history, etc.), CAT and mMRC scores, treatment during the stable stage, complications, lung function, microbiologic findings of LRT samples, chest CT findings, laboratory test results, length of hospitalization or ICU stay, hospitalization expense, mortality during hospitalization, recurrent exacerbations within 90 days, 180 days, 1 year, and the mortality within 1 year. As for smoking history, we collected the information on whether the patient had smoked, how much he/she smoked, and how long he/she had smoked. Smoking history was defined as over 10 pack-years, regardless of current smoking status.

### Statistical Analysis

Statistical analysis was performed on Stata 14 (StataCorp., College Station, TX).

Categorical variables were presented as absolute numbers and rates or percentages. Chi-square test or Fisher's exact test was used for comparison. The Shapiro-Wilk test would evaluate the normality of continuous variables. The continuous variables with normal distribution were presented as means and standard deviations, and the Student *t*-test was performed in comparison. The non-normally distributed continuous variables were presented as medians and quartiles, and the difference between the two groups was compared using Mann-Whitney *U*-test. Logistic regression was applied to explore possible risk factors for *Aspergillus* colonization and the recurrence of exacerbations. Variables with *p* < 0.10 in the univariate analysis would enter the multivariate analysis. All tests were two-tailed, and *p* < 0.05 was considered statistically significant.

## Results

A total of 1,116 patients hospitalized for AECOPD in the retrospective cohort were screened, and 626 patients met the inclusion and exclusion criteria. Thirty-one patients with *Aspergillus* and no other fungi identified in their LRT samples and 459 with no LRT fungi were kept in the remaining cases. As the baseline characteristics were significantly different in these two groups (shown in Table 1), we adopted propensity score matching at a ratio of 1:3 (caliper = 0.05). Finally, 26 cases were included in the positive group and 72 cases in the negative group for the final analysis.

**Table 1:** Baseline characteristics of hospitalized COPD patients with and without lower respiratory *Aspergillus* colonization before and after propensity score matching.

	**Before matching**		***P*-value**	**After matching**		***P*-value**
	**Patients with *Aspergillus* colonization (*n* = 31)**	**Controls (*n* = 459)**		***Aspergillus* colonization (*n* = 26)**	**Controls (*n* = 72)**	
Age	69 (61, 80)	78 (69, 84)	0.003	70 (63, 82)	71.5 (64, 82.5)	0.545
Gender (male)	25 (80.6)	388 (84.5)	0.608	20 (76.9)	52 (72.2)	0.642
Smoking history (≥10 pack-years)	10 (32.3)	379 (82.6)	<0.001	9 (34.6)	34 (47.2)	0.267
CAT score	27.5 (21, 35)	22 (15, 27)	0.001	26 (20, 34)	25.5 (22, 29)	0.721
mMRC score			0.547			0.141
1	2 (6.5)	23 (5)		2 (7.7)	0 (0)	
2	6 (19.4)	121 (26.4)		6 (23.1)	12 (16.7)	
3	14 (45.2)	178 (38.8)		11 (42.3)	41 (56.9)	
4	3 (9.7)	23 (5)		2 (7.7)	3 (4.2)	
Lost	6 (19.4)	114 (24.8)		5 (19.2)	16 (22.2)	

*Data are presented as medians (1st quartile, 3rd quartile) or absolute numbers (%) as appropriate. COPD, chronic obstructive pulmonary disease; CAT, COPD assessment test; mMRC, modified British research council*.

### Baseline Characteristics Before and After Matching

Table 1 showed the baseline data of the *Aspergillus* positive group and *Aspergillus* negative group, including the age, gender, smoking history, and the CAT scores and mMRC scores before exacerbation, which were used to assess the patients' symptom burden during the stable stage.

Before applying propensity score matching, the age in the positive group was considerably lower than that of the negative group (69 vs. 78, *p* = 0.003), and the percentage of patients with smoking history was also dramatically lower (32 vs. 83%, *p* < 0.001). The CAT scores of the positive group were notably higher compared with the negative group (27.5 vs. 22, *p* = 0.001), which presented that COPD patients with *Aspergillus* colonization had a higher symptom burden.

The baseline characteristics of the two groups were controlled at a similar level after matching of age, gender, smoking history, and CAT score and mMRC score of stable stage, which was more conducive for us to assess the clinical characteristics and prognosis of AECOPD patients based on *Aspergillus* colonization.

### Corticosteroids Use Was Related to *Aspergillus* Colonization

Inhaled corticosteroids use in patients with positive *Aspergillus* culture during the stable phase was distinctly higher than that of negative patients (65.4 vs. 27.8%, *p* = 0.001, Table 2). Univariate logistic regression analysis indicated that inhaled corticosteroids use was a risk factor for *Aspergillus* colonization (OR = 4.911, 95% CI: 1.883-12.807, *p* = 0.001). In terms of other drugs and treatment methods, we found no statistical differences. There was no remarkable difference in the prevalence of comorbidities, either.

**Table 2:** Treatment during the stable stage, comorbidities, and airflow limitation of hospitalized COPD patients with or without *Aspergillus* colonization.

	**Patients with *Aspergillus* colonization (*n* = 26)**	**Controls (*n* = 72)**	***P*-value**
**Treatment during stable stage**			
SABA	13 (50)	26 (36.1)	0.215
SAMA	3 (11.5)	11 (15.3)	0.754
LABA	0 (0)	3 (4.2)	0.563
LAMA	6 (23.1)	20 (27.8)	0.642
Inhaled corticosteroids (including ICS/LABA and ICS/LABA + LAMA)	17 (65.4)	20 (27.8)	0.001
Oral corticosteroids	1 (3.8)	1 (1.4)	0.452
Theophylline	4 (15.4)	16 (22.2)	0.458
Mucolytics	4 (15.4)	4 (5.6)	0.203
Home oxygen therapy	7 (26.9)	27 (37.5)	0.331
Home ventilator	1 (3.8)	11 (15.3)	0.173
**Comorbidities**			
Patients with complications	17 (65.4)	35 (48.6)	0.142
OSAS	1 (3.8)	1 (1.4)	0.462
Hypertension	14 (53.8)	26 (36.1)	0.115
Left ventricular dysfunction	3 (11.5)	11 (15.3)	0.754
Coronary artery disease	4 (15.4)	17 (23.6)	0.481
Cerebral apoplexy	1 (3.8)	1 (1.4)	0.462
Diabetes	2 (7.7)	12 (16.7)	0.342
**Post-bronchodilator spirometry**			
FEV_1_% pred (%)	32.05 (25.2, 58.15)	42.3 (26.8, 52.4)	0.817
FEV_1_/FVC (%)	48.63 ± 12.65	51.72 ± 10.51	0.460

*Data are presented as means ± SD, medians (1st quartile, 3rd quartile), or absolute numbers (%) as appropriate. COPD: chronic obstructive pulmonary disease; SABA, short-acting beta_2_-agonists; SAMA, short-acting muscarinic antagonists; LABA, long-acting beta_2_-agonists; LAMA, long-acting muscarinic antagonists; OSAS, obstructive sleep apnea syndrome; ICS, inhaled corticosteroids; FEV_1_, forced expiratory volume in one second; FEV_1_/FVC, forced expiratory volume in one second/forced vital capacity*.

The airflow limitation was assessed by forced expiratory volume in one second (FEV_1_)% predicted and forced expiratory volume in one second/forced vital capacity (FEV_1_/FVC) post-bronchodilators recorded in stable stage. FEV_1_% predicted and FEV_1_/FVC values of the two groups after matching showed no difference. The FEV_1_/FVC values were <70%, which met the diagnostic criteria of COPD and both groups had severe airflow obstruction.

Table 3 summarized the examination results of AECOPD patients on admission. Laboratory test results showed a difference between patients with LRT *Aspergillus* colonization and controls in the number and percentage of eosinophils (Eosinophil count: 33.21 vs. 70.4, *p* = 0.030; Eosinophils%: 0.3 vs. 0.7, *p* = 0.019). Accordingly, there was a trend of an increase in neutrophil counts and percentage in the *Aspergillus* colonization group on admission, which could be explained by the bacterial infection which served as the trigger of exacerbations. No significant difference was identified in other clinical characteristics. No laboratory results on admission were found to be a risk factor or protective factor of LRT *Aspergillus* colonization.

**Table 3:** Clinical characteristics and treatments of COPD patients on admission with or without *Aspergillus* colonization.

	**Patients with *Aspergillus* colonization (*n* = 26)**	**Controls (*n* = 72)**	***P*-value**
**Laboratory findings**			
Leucocyte count (×10^9^/L)	7.66 (5.6, 11.19)	7.89 (5.8, 9.94)	0.964
Neutrophil count (×10^9^/L)	6018 (4250, 9466)	5700 (4087, 8159)	0.525
Neutrophils% (%)	81.55 (72.7, 85.5)	75 (65, 82.9)	0.069
Eosinophil count (×10^9^/L)	33.21 (0, 42.6)	70.4 (10.8, 188.6)	0.030
Eosinophils% (%)	0.3 (0, 0.71)	0.7 (0.1, 2.6)	0.019
CRP (mg/dL)	16.85 (11.6, 34)	12.4 (3.3, 51.5)	0.326
Confirmed bacterial infection	4 (15.4)	10 (13.9)	1.000
**Chest CT findings**			
Patients with imaging findings	16 (61.5)	42 (58.3)	0.776
Consolidation	2 (7.7)	3 (4.2)	0.606
Patchy shadows	13 (50)	33 (45.8)	0.715
Multilobar lesions	6 (23.1)	15 (20.8)	0.811
Bilateral lesions	7 (26.9)	35 (48.6)	0.055
Pleural effusion	2 (7.7)	10 (13.9)	0.507
**Treatment during hospitalization**			
Mechanical ventilation	6 (23.1)	25 (34.7)	0.274
Invasive mechanical ventilation	5 (19.2)	23 (31.9)	0.219
Non-invasive mechanical ventilation	1 (3.8)	3 (4.2)	1.000

*Data are presented as medians (1st quartile, 3rd quartile) or absolute numbers (%) as appropriate. COPD, chronic obstructive pulmonary disease; CRP, C-reactive protein*.

### Patients With *Aspergillus* Colonization Were at a High Risk of Relapse of AECOPD

Table 4 presented the clinical outcomes of the patients during hospitalization and within 1 year after discharge. We did not find notable differences in length of hospitalization, length of ICU stays, hospitalization costs, and in-hospital mortality. Compared with those *Aspergillus* cultured-negative, the rate of recurrent exacerbation within 90 days of the *Aspergillus* positive group was significantly higher (19.2 vs. 4.2%, *p* = 0.029). The recurrence rate within 180 days was obviously higher, too (23.1 vs. 4.2%, *p* = 0.010). There was no considerable difference in the rate of recurrence of AECOPD within 1 year. The all-cause 1-year mortality was higher in *Aspergillus* positive group than the negative group (11.5 vs. 0.0%, *p* = 0.017).

**Table 4:** Prognosis of hospitalized COPD patients with or without *Aspergillus* colonization.

	**Patients with *Aspergillus* colonization (*n* = 26)**	**Controls (*n* = 72)**	***P*-value**
Length of hospital stay (days)	12 (7,22)	13 (10,15)	0.759
ICU admission	2 (7.7)	9 (12.5)	0.722
Hospitalization expense (RMB)	17,290 (12,601, 23,049)	17,110 (13,204, 21,348)	0.728
Mortality during hospitalization	1 (3.8)	1 (1.4)	0.462
Recurrent exacerbations within 90 days	5 (19.2)	3 (4.2)	0.029
Recurrent exacerbations within 180 days	6 (23.1)	3 (4.2)	0.010
Recurrent exacerbations within 1 year	9 (34.6)	39 (54.2)	0.087
Mortality within 1 year	3 (11.5)	0 (0)	0.017

*Data are presented as medians (1st quartile, 3rd quartile) or absolute numbers (%) as appropriate. COPD, chronic obstructive pulmonary disease; ICU, intensive care unit*.

Tables 5, 6 manifested the results of logistic regression analysis. Univariate analysis proved that *Aspergillus* colonization was a risk factor for relapse of acute exacerbation within 90 days and 180 days (90 days: OR = 5.476, 95% CI: 1.207-24.849, *p* = 0.028; 180 days: OR = 6.9, 95% CI: 1.582-30.087, *p* = 0.010). Besides, pleural effusion in chest CT findings was also a risk factor of recurrent exacerbations in COPD patients (90 days: OR = 5.4, 95% CI: 1.103-26.438, *p* = 0.037; 180 days: OR = 4.444, 95% CI: 0.945-20.893, *p* = 0.059). Multivariate analysis agreed with the results of univariate analysis. Variables associated with the relapse of acute exacerbation were *Aspergillus* colonization (90 days: OR = 8.661, 95% CI: 1.496-50.159, *p* = 0.016; 180 days: OR =10.723, 95% CI: 1.936-59.394, *p* = 0.007) and pleural effusion (90 days: OR = 9.793, 95% CI: 1.470-65.237, *p* = 0.018; 180 days: OR = 8.791, 95% CI: 1.317-58.689, *p* = 0.025).

**Table 5:** Univariate and multivariate logistic analysis for relapse of acute exacerbation in COPD patients within 90 days after discharge.

	**Univariate logistic analysis**			**Multivariate logistic analysis**		
	**OR**	**95% CI**	***P*-value**	**OR**	**95% CI**	***P*-value**
*Aspergillus* colonization	5.476	1.207-24.849	0.028	8.661	1.496-50.159	0.016
FEV1% pred (%)	0.928	0.783-1.101	0.391			
FEV_1_/FVC (%)	0.945	0.769-1.162	0.592			
Neutrophils% (%)	1.022	0.955-1.094	0.527			
Eosinophils% (%)	0.715	0.369-1.387	0.321			
Patients with imaging findings	2.192	0.419-11.462	0.352			
Consolidation	3.071	0.301-31.341	0.344			
Patchy shadows	1.992	0.449-8.840	0.365			
Multilobar lesions	0.500	0.058-4.307	0.528			
Bilateral lesions	0.785	0.177-3.484	0.750			
Pleural effusion	5.4	1.103-26.438	0.037	9.793	1.470-65.237	0.018
Length of hospital stay	1	0.898-1.114	1.000			

*COPD, chronic obstructive pulmonary disease; FEV_1_, forced expiratory volume in one second; FEV_1_/FVC, forced expiratory volume in one second/forced vital capacity*.

**Table 6:** Univariate and multivariate logistic analysis for relapse of acute exacerbation in COPD patients within 180 days after discharge.

	**Univariate logistic analysis**			**Multivariate logistic analysis**		
	**OR**	**95% CI**	***P*-value**	**OR**	**95% CI**	***P*-value**
*Aspergillus* colonization	6.9	1.582-30.087	0.010	10.723	1.936-59.394	0.007
FEV1% pred (%)	0.844	0.687-1.037	0.107			
FEV_1_/FVC (%)	0.852	0.702-1.034	0.106			
Neutrophils% (%)	1.010	0.950-1.075	0.741			
Eosinophils (%)	0.656	0.328-1.310	0.232			
Patients with imaging findings	2.608	0.513-13.265	0.248			
Consolidation	2.656	0.264-26.709	0.407			
Patchy shadows	2.45	0.576-10.418	0.225			
Multilobar lesions	0.431	0.051-3.657	0.441			
Bilateral lesions	1.074	0.270-4.269	0.920			
Pleural effusion	4.444	0.945-20.893	0.059	8.791	1.317-58.689	0.025
Length of hospital stay	0.962	0.855-1.081	0.513			

*COPD, chronic obstructive pulmonary disease; FEV_1_, forced expiratory volume in one second; FEV_1_/FVC, forced expiratory volume in one second/forced vital capacity*.

## Discussion

### *Aspergillus* Colonization Predict Poor Outcomes of AECOPD

We investigated a large sample of more than 1,000 patients with severe exacerbation of COPD from eight centers and collected abundant information on the stable stage, acute exacerbation stage, clinical outcomes, and prognosis. While other researchers focused on the risk factors and clinical manifestations of COPD patients colonized or infected by *Aspergillus*, we were the first to concentrate on the prognosis of COPD patients after hospitalization discharge. We established three follow-up periods of 90 days, 180 days, and 1 year to observe the short-term and long-term prognosis, which was a new attempt in similar researches. The results of this study suggested that *Aspergillus* colonization is associated with poor clinical outcomes of hospitalized AECOPD, including higher acute exacerbation rates within 90 days, 180 days, and higher mortality within 1 year after discharge.

We speculated that the worse prognosis is attributed to AH. Firstly, COPD patients are more likely to have an allergic reaction to *Aspergillus* than those without the disease. A prospective multicenter study demonstrated that COPD had a high frequency of sensitization in various allergens, including *Aspergillus* (13). Agarwal et al. found AH in 8.5% (17 in 200) COPD patients, while none in healthy volunteers (10). Secondly, COPD patients with *Aspergillus* were at higher levels of specific-IgE (14), related to AH. A study of *Aspergillus* sensitization and asthma's relationships indicated that *Aspergillus*'s long-term colonization is related to sensitization (15). For COPD patients, it is also possible that persistent *Aspergillus* colonization has a stimulating effect on AH. Thirdly, AH can lead to decreased lung function and severe respiratory symptoms (11, 12). Tiew et al. found that fungal sensitization was associated with the frequency of exacerbations (13). Moreover, another study suggested that the cluster characterized by *Aspergillus, Curvularia*, and *Penicillium* had increased exacerbations and higher mortality than another cluster with *Saccharomyces* (14). These findings are consistent with ours, indicating that it is long-term colonization of *Aspergillus* in the airways of COPD patients that cause sensitization and make patients more susceptible to acute exacerbations under specific stimulation.

Furthermore, researchers found that sensitization to recombinant antigens of *Aspergillus* increases the risk for bronchiectasis in COPD patients (16). And a study published recently identified that the number of fungal interactions is increased in COPD patients with very frequent exacerbations (exacerbate more than three times per year) (14). To some extent, these findings support our conclusion that *Aspergillus* causes a worse prognosis in COPD patients.

However, the molecular mechanism of the effects of *Aspergillus* on acute exacerbation remains unclear. The reason for no statistical difference in 1-year mortality is also waiting to explore. Would the intensity of AH fluctuate over time and result in the difference between short-term and long-term outcomes? Is it possible to determine which COPD patients are susceptible to AH based on specific characteristics? Is it able to help with treatment strategies by measuring total-IgE or specific-IgE? These are the keys to future study.

Also, we found that the eosinophil count and eosinophils% on admission in *Aspergillus* positive group were lower than those in the control group, which might be explained by the bacterial infection which served as the trigger of exacerbations.

### The Risk Factors of *Aspergillus* Colonization

We observed that *Aspergillus* was more likely to colonize in patients treated with inhaled corticosteroids during the stable stage. This observation is consistent with the study of Tong et al. (6), who found that the proportion of patients who inhale high doses of corticosteroids during the stable stage in the *Aspergillus* colonization group was higher than that in the control group. Another study also indicated that patients with *Aspergillus* cultured positive had a higher inhaled corticosteroid dose during the stable stage compared with those cultured negative (12). The use of corticosteroids reduces the anti-fungal capacity of macrophages in the airways, inhibits the production of proinflammatory cytokine and chemokine (17, 18). In general, corticosteroids inhibit the ability of immune systems to eliminate *Aspergillus* conidia and promote the growth of *Aspergillus* (19). So, the use of inhaled corticosteroids is responsible for *Aspergillus* colonization.

The study of Tong et al. suggested that smoking history should be a risk factor for *Aspergillus* colonization (6). Chronic tobacco smoke can damage the function of cilia, reduce the removal rate of *Aspergillus* in the airways, and adjust the microbial environment to be suitable for *Aspergillus*'s growth. In our baseline data before matching, the lower percentage of smokers in the *Aspergillus* positive group should be due to selection bias.

The genetic factor is also a possible risk factor for *Aspergillus* colonization. It is showed that genetic mutations could affect protein levels in the extracellular matrix and promote the adhesion of *Aspergillus* conidia. Moreover, affected by genetic factors, some patients might show a stronger inflammatory response to the conidia of *Aspergillus* (20).

### Strengths and Limitations

This study was the first retrospective multicenter study on the impact of LRT *Aspergillus* colonization on the early relapse long-term prognosis of AECOPD. Another strength is propensity score matching. By matching control age, sex, smoking history, and severity of symptoms before acute exacerbation, this study minimized the individual differences in patients and obtained the data of impacts of *Aspergillus* colonization on the prognosis.

However, our study had several limitations. Firstly, this was a retrospective study. We could not confirm whether *Aspergillus* colonization was acquired during the stable stage or after the onset of the exacerbation. We only proved the relationship between the LRT *Aspergillus* colonization and outcome variables, like other available studies on this topic (6). Secondly, the positivity rate of *Aspergillus* culture was quite low (6%, 31/490), as we excluded the patients who received antifungal therapy, who should have positive results of *Aspergillus* culture. We didn't process the sputum samples to obtain homogenized sputum or sputum plugs, and we didn't use high-volume culture or quantitative real-time polymerase chain reaction to detect *Aspergillus*, which might be responsible for the low positive rate (21–23). We plan to perform a prospective study and adopt more sensitive methods to detect *Aspergillus*. Thirdly, data collected from medical records could not help us screen out the patients with AH through hypersensitivity skin testing or IgE determination. We intend to verify the causal relationship and explore the impacts of AH in the prospective study. Besides, we do not have a comprehensive microbiological test result, which may have some influence on the conclusion. It is reported that changes in the microbiome of COPD patients' airways may be related to the risk of exacerbations (14, 24). After various stimulations induce an inflammatory response, the airway environment would be conducive to the growth of certain microorganisms. This leads to the disturbance and imbalance of the airway microbiome homeostasis, causing further airway inflammation and forming a vicious circle (25, 26). For instance, several studies have confirmed that COPD patients who are positive for *Pseudomonas aeruginosa* culture have a higher risk of acute exacerbations and higher mortality (27, 28). More comprehensive detection of the microbiome in the airway will be taken in our future prospective study.

In conclusion, we presented that *Aspergillus* colonization would lead to the relapse of exacerbation in hospitalized AECOPD patients in a short period. We should pay attention to the *Aspergillus* cultured in LRT samples. But the mechanisms and the adjustment of clinical strategies still require further studies.

## Data Availability Statement

The original contributions presented in the study are included in the article/supplementary material, further inquiries can be directed to the corresponding author/s.

## Ethics Statement

The studies involving human participants were reviewed and approved by the Ethics Committee of Zhongshan Hospital of Fudan University (Approval notice number: B2015-119R2017-108). Written informed consent for participation was not required for this study in accordance with the national legislation and the institutional requirements.

## Author Contributions

Y-xW collected and analyzed the data and wrote the manuscript. Y-hZ screened the patients and checked the data. Q-jC, YH, Z-yB, X-yJ, X-wG, C-lT, W-pH, J-qH, W-qW, and F-yZ contributed to the data collection. JZ designed, supervised the study, and revised the manuscript. All authors contributed to the manuscript, read, and approved the submitted version.

## Conflict of Interest

The authors declare that the research was conducted in the absence of any commercial or financial relationships that could be construed as a potential conflict of interest.
